# Circadian rhythms of arousal in parent–child interaction: a 24-hour co-regulation process

**DOI:** 10.1093/cdpers/aadaf016

**Published:** 2026-01-29

**Authors:** Chase J Boyer, Leah C Hibel

**Affiliations:** Department of Human Ecology, University of California, One Shields Ave, Davis, CA 95616, United States; Department of Human Ecology, University of California, One Shields Ave, Davis, CA 95616, United States

**Keywords:** circadian rhythms, caregiving, sleep–wake cycles, dynamic systems theory, transactional models

## Abstract

Caregivers play a critical role in regulating infants’ arousal and synchronizing biological rhythms with environmental cycles. In this article, we examine how caregiving behaviors shape the development of circadian rhythms in early childhood, focusing on the integration of sleep–wake cycles and the function of the hypothalamic–pituitary–adrenal axis. We propose a framework that is grounded in dynamic systems theory and transactional models, and that highlights the bidirectional interactions between caregivers and infants in establishing a cohesive 24-hour regulatory system. We also explore the cascading effects of disruptions in sleep and stress regulation, emphasizing how responsive caregiving supports adaptive outcomes while inconsistent caregiving may lead to dysregulation.

Young children rely on their caregivers to externally regulate states of arousal (Feldman, 2017; Gunnar & Donzella, 2002). In early childhood, the stress response system is largely dependent on caregivers to soothe and calm heightened emotional and physiological arousal. Similarly, infants are unable to sleep through the night and depend on their caregivers to meet needs such as feeding and comfort (Sadeh et al., 2009).^1^ Through these ongoing responsive interactions, caregivers and infants co-regulate biological rhythms to environmental cycles, supporting social, emotional, and physiological development (Propper & Moore, 2006). While much research has focused on either sleep or stress regulation, few studies have examined how caregiving shapes the integration of the two within a cohesive 24-hour circadian system (McLaughlin et al., 2022; Scher et al., 2010; Tuladhar et al., 2021).

Understanding the interdependence of sleep and stress systems aligns with broader developmental theories, such as dynamic systems theory, which emphasizes the interdependence of biological, behavioral, and environmental systems over time (Thelen & Smith, 1994, 2006). This 24-hour circadian framework describes how disruptions in one system can cascade into others, affecting long-term developmental outcomes. Furthermore, transactional models highlight the bidirectional influences between young children and their environment (e.g., caregivers, culture, community), situating circadian regulation as both a product of and a contributor to developmental trajectories (Sameroff, 2010). In this article, we propose a framework to help understand how caregivers scaffold the integration of infants’ sleep and stress response systems into a unified circadian arousal system.

## The development of circadian rhythms in early life

Circadian rhythms are endogenously generated biological processes that follow an approximately 24-hour cycle, aligning physiological and behavioral functions with environmental time (Reppert & Weaver, 2002). From an evolutionary perspective, these rhythms allow organisms to adapt to daily environmental changes (Mistlberger, 2005). In humans, a cluster of neurons in the anterior hypothalamus known as the suprachiasmatic nucleus (SCN) serves as the master circadian pacemaker, regulating the timing of biological processes across multiple systems (Saper et al., 2005). The SCN receives direct input from the retina about light and darkness, and in turn, coordinates peripheral clocks in other brain regions and organs through hormonal and neural signaling (Buijs & Kalsbeek, 2001). Although the SCN is functional at birth, it is immature and not yet fully entrained (i.e., synchronized) to environmental light–dark cycles. Infants’ biological rhythms are not yet stable and require external scaffolding through environmental cues provided by caregivers (Frank, 2020). Consistent light exposure, feeding, and soothing routines help entrain the SCN to external time cues, facilitating the consolidation of circadian systems.

Furthermore, these caregiver-driven feeding and sleeping routines reflect the sociocultural practices of the family. While the biological foundations of circadian rhythms are universal, their expression also reflects the sociocultural practices of their context (Jenni & O’Connor, 2005). In terms of caregiving, individualistic cultures (e.g., those that dominate countries like the United States and the United Kingdom) emphasize independence autonomy, whereas collectivistic cultures (e.g., those in countries such as Japan, India, and many Latin American cultures) emphasize interdependence and social harmony (Tamis-LeMonda et al., 2008). These are general patterns and do not represent all families within a cultural group. Also, there are limitations in using the broad dichotomies of East–West or individualistic–collectivistic culture, and many scholars have urged greater attention to within-culture variability (Vignoles et al., 2016). Caregiving practices around sleep may reflect broader cultural values, with some Western contexts emphasizing independence and structured sleep routines, while others emphasize interdependence and fluid temporal rhythms (Barry & McKenna, 2022; Mindell et al., 2010). For example, Dutch caregiving emphasizes rest, regulation, and cleanliness as central to infants’ routines that lead to more stable circadian rhythms (van Schaik et al., 2020). In contrast, cultures emphasizing flexibility and responsiveness may organize infants’ sleep routines around social engagement or work patterns rather than strict time-based schedules (Yovsi & Keller, 2007). These cultural differences in caregiving routines, sleep environments, and regulatory expectations shape the developmental trajectory of circadian regulation.

### Development of the HPA axis diurnal rhythm

The hypothalamic–pituitary–adrenal (HPA) axis is a central component of the body's arousal and stress response systems. The product of the HPA axis, the hormone cortisol, supports metabolic regulation, energy mobilization, and adaptation to stressors (Gunnar & Quevedo, 2007). Cortisol has both baseline and reactive functions. Under typical conditions, cortisol release follows a distinct diurnal rhythm, peaking within 30–45 minutes of waking (i.e., the cortisol awakening response), and gradually declining throughout the day, reaching its lowest levels around midnight (Adam et al., 2017; Stalder et al., 2022). In contrast, cortisol can also spike acutely in response to stress, returning to baseline through a negative feedback loop. While both basal and reactive forms of cortisol release are present during infancy, the maturation of a predictable day–night rhythm unfolds gradually and is a key developmental process over the first year of life.

During the first several weeks after birth, cortisol levels in infants vary significantly and are largely responsive to internal physiological needs rather than external cues, with little evidence of a clear diurnal rhythm (Iwata et al., 2013). By 6–12 weeks, early signs of a day–night cortisol rhythm begin to appear, with modest morning increases and evening decreases (de Weerth et al., 2003). Between 3 and 6 months, the diurnal pattern of cortisol becomes more apparent as the SCN becomes more sensitive to light cues (Kervezee et al., 2024). Morning cortisol levels show a more robust peak, while the decline across the day becomes more gradual and continuous. These changes reflect the increasing synchronization between central circadian timing mechanisms and hormonal outputs, particularly the alignment of cortisol secretion with cues regulated by the SCN (Saper et al., 2005). By 6–9 months, most infants exhibit a more stable diurnal profile, with cortisol peaking within the first hour after waking and declining throughout the day to reach a trough near midnight, though timing and amplitude vary widely (Adam et al., 2017; Kervezee et al., 2024).

Overall, the development of a diurnal cortisol rhythm represents a key milestone in the regulation of early arousal. It reflects the integration of central circadian timing mechanisms with peripheral hormonal outputs, supporting infants’ capacity to transition between physiological states of rest and activation across the 24-hour cycle (Kervezee et al., 2024). As infants move through the first year of life, the rhythm becomes increasingly stable yet remains sensitive to environmental input (Wong et al., 2022). This period of refinement marks a shift from the basic establishment of the cortisol rhythm to its growing coordination with the sleep–wake cycle, which follows a parallel trajectory of circadian alignment. This emerging rhythmicity does not follow a uniform trajectory across all infants, but instead reflects a dynamic process of neuroendocrine maturation, shaped by intrinsic timing and contextual variability.

### Development of sleep–wake circadian rhythms

Sleep is a foundational physiological process that supports infants’ growth, brain development, and emotional regulation (Dahl, 1996; El-Sheikh et al., 2007). Unlike in adulthood, sleep in infancy is highly fragmented and polyphasic, reflecting an immature neurological system that is still adapting to environmental cues. Over the first year, infants experience rapid changes in both the structure (i.e., the distribution of active versus quiet sleep, organization of sleep stages) and the timing (i.e., when and how long they sleep) of sleep (Iglowstein et al., 2003). Infant’s transition from short, frequent episodes of sleep scattered across the 24-hour period to increasingly consolidated patterns of nighttime sleep and daytime naps, gradually aligning with the day–night cycle (Burnham et al., 2002; Henderson et al., 2011). The emergence of a circadian rhythm in sleep depends on the maturation of key brain regions, including the brainstem, thalamus, and hypothalamus, which regulate arousal, sleep-state transitions, and the alignment of timing with the 24-hour cycle (Lokhandwala & Spencer, 2022). In particular, the SCN modulates the timing of sleep–wake cycles by synchronizing internal rhythms with environmental light cues (Wong et al., 2022). As these neural systems mature, infants begin to show more predictable sleep–wake patterns that reflect circadian organization.

In the newborn period (from birth to 2 months), the timing of sleep is driven by internal needs such as hunger, occurring in short, fragmented episodes, with minimal distinction between nighttime and daytime sleep (Sadeh et al., 2009). By 3–6 months, a clearer day–night pattern emerges, with longer nighttime sleep and more consistent daytime napping (Iglowstein et al., 2003). This shift toward more consistent day–night sleep patterns is considered a hallmark of early circadian entrainment (Henderson et al., 2011). Between 6 and 12 months, sleep consolidates into longer nighttime bouts and two to three structured naps per day (Mindell et al., 2016). However, this structured nap pattern typically occurs in infants raised in cultures that promote scheduled solitary sleep. In many collectivistic cultures, where infants co-sleep or are worn on their caregivers’ bodies during the day, daytime sleep may occur in shorter, more variable episodes, and napping may not happen on a fixed schedule (Yovsi & Keller, 2007). During the second year of life, total sleep duration decreases due to fewer daytime naps, with consolidation complete once children have stopped napping altogether at around 5–7 years (Acebo et al., 2005; Mindell et al., 2016).

Sleep regulation varies widely across infants, particularly in terms of night wakings. Although the ability to *sleep through the night* is often used as a developmental benchmark of self-regulated sleep, sleep is inconsistently defined in this term and does not mean the absence of night wakings (Henderson et al., 2011). Rather, self-regulated sleep occurs when infants can return to sleep independently after naturally occurring arousals. Part of this variation in infants’ sleep regulation is due to culturally determined expectations of infants’ independence and parenting behaviors toward these expectations (Barry, 2021). For example, in some cultures (e.g., Japan), on-demand through-the-night feeding, co-sleeping, and regular parental external regulation are the norm, while in other cultures (e.g., mainstream U.S. culture), independent sleeping and minimal intervention are expected (Ball, 2007). In a review of sleep indicators in early childhood (Galland et al., 2012), across countries, children showed a decline in night wakings by age 2, but compared with other sleep parameters, night wakings varied the most. During the second year of life, night wakings continue to decrease as most infants learn to self-soothe back to sleep, representing a shift from physiologically driven fragmentation to circadian consolidation (Burnham et al., 2002; Pecora et al., 2022).

### Integration of sleep and HPA axis functioning

As infants develop, sleep–wake cycles and HPA axis activity become increasingly synchronized components of a unified circadian regulatory system. This integration involves specific neurobiological mechanisms centered on the SCN, which coordinates timing signals to both systems (Wong et al., 2022). The SCN regulates melatonin production in the pineal gland, promoting sleep onset as cortisol levels decline, while also mediating the cortisol awakening response that facilitates morning wakefulness (Oster et al., 2017). This coordinated control creates multiple pathways through which sleep–wake patterns and cortisol rhythms can influence each other's functioning, establishing a complex feedback system that matures throughout early development.

The relation between sleep–wake patterns and cortisol secretion is fundamentally bidirectional. Poor sleep can alter stress reactivity through disrupted negative feedback mechanisms in the HPA axis in adults and older children, reducing glucocorticoid receptor sensitivity in the hippocampus and hypothalamus, impairing the ability of cortisol to suppress its own production (van Dalfsen & Markus, 2018). This disruption leads to impaired negative feedback control, allowing cortisol production to continue when it would usually decrease, and affecting the timing and amplitude of the next day's cortisol rhythms (Buckley & Schatzberg, 2005). In studies with older children, poor sleep quality is associated with flatter morning-to-evening cortisol slopes (El-Sheikh et al., 2008) and disrupted cortisol awakening responses (Räikkönen et al., 2010). Despite ample evidence for a bidirectional association between the HPA axis and sleep systems in childhood and beyond, few studies have investigated this phenomenon in infants.

In one study, researchers found bidirectional associations between poor sleep and elevated cortisol in infants with colic (Brand et al., 2011). In other studies, among toddlers, fragmented sleep was associated with higher cortisol awakening responses (Scher et al., 2010), while stable awakening response related to better sleep (Bright et al., 2014). The bidirectionality of these effects is also evident in concentrations of cortisol in hair, demonstrating the impact of sleep on chronic activation of the HPA axis in infancy (Flom et al., 2017; Tuladhar et al., 2021). Indeed, in both of these studies (Flom et al., 2017; Tuladhar et al., 2021), later sleep onset and shorter sleep duration were associated with greater concentrations of cortisol in infants’ hair, but more typical and regulated (i.e., steeper) diurnal slopes were related to longer sleep and fewer night wakings. Taken together, these studies lay the foundation for researchers to explicitly examine the pattern of bidirectionality between the developing sleep–wake circadian system and the HPA axis system.

This emerging circadian integration follows a developmental trajectory characterized by increased temporal coordination. By 3–4 months, initial coordination of these systems emerges, with early signs of a diurnal cortisol system beginning to synchronize with the emerging day–night sleep organization (de Weerth et al., 2003). During this period, longer consolidated sleep episodes start to coincide with the natural evening decline in cortisol levels, though this alignment remains inconsistent and is easily disrupted by environmental and caregiving factors (Wong et al., 2022). Between 6 and 12 months, as diurnal cortisol patterns become more defined and nighttime sleep consolidates, these systems show stronger temporal coordination, with morning cortisol increases aligning with waking and evening cortisol decreases facilitating sleep onset (Flom et al., 2017; Kervezee et al., 2024). By the end of the second year, this coordination typically becomes more robust and resistant to minor disruptions, though it is still more plastic and vulnerable to environmental influences in toddlers than it is in older children and adults (Saridjan et al., 2017).

It is important to distinguish between normative developmental variability and problematic dysregulation. Temporary circadian disruptions are common during developmental transitions or minor illnesses and typically resolve with consistent routines (Sadeh et al., 2009). In contrast, sleep disruptions alongside atypical cortisol patterns may indicate a more problematic trajectory that could affect broader regulatory capacities (Saridjan et al., 2017). However, the transition from temporary to persistent dysregulation often occurs gradually through cumulative effects of repeated daily disruptions (e.g., Flom et al., 2017; Tuladhar et al., 2021). When day-to-day sleep and cortisol misalignments recur frequently, they can begin to alter the sensitivity of regulatory systems and establish increasingly entrenched patterns (Berry et al., 2012; McEwen, 2007). These repeated disruptions can create self-reinforcing feedback loops in which fragmented sleep disrupts cortisol regulation the next day, leading to higher levels of evening cortisol that interfere with subsequent sleep. With each iteration of this cycle, physiological adaptations can occur, including changes in glucocorticoid receptor sensitivity and alterations in sleep architecture (i.e., the pattern of different sleep stages) that make the system increasingly vulnerable to further disruptions (Lokhandwala & Spencer, 2022). Over time, repeated disruptions may lead to persistent dysregulation characterized by chronic sleep fragmentation and atypical cortisol patterns, potentially affecting multiple developmental domains including emotional regulation and cognitive functioning. This integration represents a critical developmental process that should be explored in the context of caregiver–infant relationships.

## Caregiver–child interactions and regulation of infants’ circadian rhythms

We propose a framework that extends beyond the well-established role of caregivers as external regulators of infants’ arousal (Feldman, 2017; Gunnar & Donzella, 2002), to indicate that caregivers play a critical and unique role in facilitating the coordination and integration of infants’ sleep and HPA axis systems into a cohesive circadian regulatory network. This integrative function represents a distinct aspect of caregiving that has received limited attention in developmental research. Next, we describe how caregivers promote synchronization between these systems through consistent routines, support for transitions between sleep states, and balanced regulation of arousal. We then explore how this integration process operates bidirectionally and initiates developmental cascades, with implications beyond either sleep or HPA axis regulation alone.

### Caregivers as integrators of circadian systems

Caregivers can facilitate circadian integration of stress and sleep systems through several pathways. First, they help establish a temporal consistency through daily routines that simultaneously entrain both sleep–wake cycles and activity–rest patterns of cortisol secretion (Spruyt et al., 2008). By maintaining regular schedules for feeding, sleep, and social interaction, caregivers provide predictable environmental cues that help align multiple biological rhythms with the 24-hour day (LeBourgeois et al., 2013). Second, caregivers manage the synchrony between sleep and the functioning of the HPA axis through moment-to-moment regulation of infants’ arousal states (Feldman, 2012; Laurent et al., 2016). When caregivers respond sensitively to infants’ cues, they help prevent excessive HPA activation that could disrupt sleep (Philbrook, 2022; Tuladhar et al., 2021) while also preventing sleep disruptions that could dysregulate cortisol patterns (Philbrook et al., 2014). This balanced regulation across both systems simultaneously is distinct from regulating either system independently. Third, caregivers create transitional bridges between different arousal states, helping infants navigate the complex physiological shifts between sleep and wakefulness (Teti et al., 2010; Tikotzky, 2017). How well parents support the shift from alert wakefulness to sleep onset appears to influence both sleep architecture and the decline of nighttime cortisol (Philbrook & Teti, 2016). Thus, emotionally available bedtime interactions characterized by sensitive responses to infants’ distress may facilitate this coordinated transition in both systems simultaneously.

Infants’ individual characteristics inform these integrative functions. Infants with more reactive temperaments require more responsive support from caregivers to achieve better sleep (Bernier et al., 2014; Camerota et al., 2019; Laurent et al., 2016). In these cases, caregivers may be attuned to providing the consistent external regulation that helps coordinate sleep–wake patterns and HPA axis functioning. Based on previous theoretical and empirical work, we propose that when parents successfully provide this support for infants with higher reactivity or innate sleep regulation challenges, these infants may achieve regulatory outcomes similar to those of infants predisposed to more regulated sleep–wake patterns or lower physiological reactivity (Belsky & Pluess, 2009; Jian & Teti, 2016).

### Cross-system cascades and developmental implications

The most compelling evidence for caregivers’ role in circadian coordination comes from studies of developmental cascades that cross between sleep and HPA systems. These cross-system effects contribute to broader developmental cascades that unfold over time. Researchers have established that disruptions in sleep regulation predict later cognitive and emotional difficulties (El-Sheikh et al., 2007; Hoyniak et al., 2019), while early HPA axis functioning separately predicts developmental outcomes across multiple domains (Berry et al., 2012). Similarly, sensitive caregiving predicts both better sleep patterns (Kim & Teti, 2014) and more adaptive HPA axis regulation (Gunnar & Hostinar, 2015). Although few studies have directly examined the integrated function of these circadian systems in relation to caregiving, the interconnections among them suggest that short-term cascades among sleep, cortisol regulation, and caregiving likely contribute to long-term developmental trajectories (Camerota et al., 2019). This points to a compelling developmental pathway through which momentary parent–child interactions scaffold the coordination of physiological systems, which in turn supports broader developmental outcomes that extend far beyond immediate regulatory functioning.

Parenting strategies around infants’ sleep tend to reflect broader cultural values and living arrangements rather than fixed cultural divisions. For instance, some caregivers emphasize independent sleep routines, while others prioritize proximity and responsiveness through shared sleep arrangements (Mindell et al., 2010). These perspectives reflect both cultural values and developmental timing. During early infancy, co-sleeping or room sharing may facilitate rapid caregiver responses and support co-regulation (Barry, 2019). Indeed, the American Association of Pediatrics recommends room sharing (but not bed-sharing) for the first 6 months of infancy (Moon et al., 2022). In cultures that emphasize independence, co-sleeping is viewed as interfering with the development of self-regulation and is often done reactively, whereas in cultures in which co-sleeping is more common, it is less disruptive to infants’ sleep (Barry & McKenna, 2022). The transition from co-regulation to self-regulation is likely gradual and probably involves periods of dysregulation as infants learn new skills.

## Implications for research

Researchers should operationally define circadian coordination in early life as the temporal alignment and magnitude of association between sleep–wake patterns and diurnal cortisol rhythms. Coordination can be measured as (1) the temporal association between individual shifts in sleep–wake patterns and diurnal cortisol slopes, (2) the synchrony between sleep onset and evening cortisol decline, and (3) the correlation between individual differences in sleep efficiency and the magnitude of cortisol awakening response. An integrated circadian system would appear as a positive association between sleep–wake patterns and HPA axis functioning, with poorer sleep associated with greater HPA activation or altered diurnal patterns without changing output. However, central to this framework is that the magnitude of this association can be moderated by the caregiving context. Measuring these operational definitions requires rigorous methods.

Researchers should use intensive longitudinal designs with ecological momentary assessment over multiple bursts to track developmental trajectories. These assessment protocols can be structured to collect from both caregivers and infants objective sleep data via actigraphy (i.e., noninvasive wrist- or ankle-worn devices) and videosomnography (i.e., noninvasive video recordings), as well as salivary cortisol sampled four times per day (at awakening, 30 minutes after wake-up, in the evening, and at bedtime) to capture the full diurnal rhythm. However, repeated daily saliva collection can be demanding for working parents and their infants. As demonstrated by studies (Flom et al., 2017; Tulahdar et al., 2021), periodically including measures of cortisol concentrations in hair can assess chronic HPA axis activation. This approach provides researchers the opportunity to capture parenting behaviors, bedtime interactions, momentary and daily caregiving stress and responsiveness, and measures of emotional availability during sleep-related caregiving.

These protocols allow researchers to explore critical questions: How do parenting behaviors facilitate or disrupt sleep-HPA coordination across development? Can caregiving patterns that promote coordination serve as targets for intervention? How do individual infant characteristics interact with parenting to shape coordination development? Researchers should also investigate how cultural values and caregiving practices shape the synchronization of sleep–wake and HPA axis rhythms, including whether culturally normative routines (e.g., co-sleeping versus solitary sleeping) predict different trajectories of circadian regulation. Addressing these questions requires examining how bedtime parenting, nighttime responsiveness, and stress management predict coordination trajectories; comparing integrated versus single-system models; testing sensitivity-focused interventions, and investigating how individual differences in infants’ reactivity moderate the effects of caregiving on integration of the circadian system.

## Conclusion

By framing sleep and HPA rhythms as components of a unified circadian regulatory system, we have offered a novel contribution to understanding how early arousal regulation unfolds across infancy. Our framework highlights how sleep and HPA axis integration shapes adaptive and maladaptive developmental trajectories. Understanding how these interconnected systems function and develop can offer insights into early developmental processes, providing a foundation for supporting resilience and adaptation in infancy and beyond.

## Supplementary Material

aadaf016_Supplementary_Data

## Footnotes

1 For the sociodemographic characteristics of the studies reviewed herein, please see Table S1 in online materials.

## Author note

The institutional review board (IRB) at University of California, Davis approved all study procedures. Correspondence concerning this article should be addressed to the first author, Leah C. Hibel.
