# Supplementary material for: Circadian rhythms of arousal in parent–child interaction: a 24-hour co-regulation process
Source: Child Dev Perspect. 2026 Jan 29;20(1):47–54. doi: 10.1093/cdpers/aadaf016 (PMC13046071; doi:10.1093/cdpers/aadaf016)
Supplement: aadaf016_Supplementary_Data [file aadaf016_supplementary_data.pdf]

Supplemental Table 1

Sociodemographic Characteristics of Studies Cited in the Article, Alphabetized by First Author's Last Name

Authors	Year	Country	Race / Ethnicity	Sample Size	Sex	SES	Urban/Rural	Age
Acebo et al.	2005	United States	91% White	169	50.3% Girl	Higher SES	Urban	169 normal healthy children in 7 age groups (12, 18, 24, 30, 36, 48, and 60 months old)
Adam et al.	2017	NA	NA	80 Studies; 179 effect sizes	NA	NA	NA	infant/ toddler; School-age children; Adults; Older Adults
Bai et al	2022	United States	86% White	167 Mother-infant dyads	51.6% Girl	Middle Class: 70% College degree	Rural and semirural communities	12-, 18-, 24-months
Bai et al	2019	United States	86% White	142 Mother-infant dyads	53% Girl	Middle Class: 70% College degree	Rural and semirural communities	1-, 3-, 6-months
Bernier et al.	2014	Canada	N/A	63 Mother-infant dyads	27 girls and 36 boys	Diverse, mostly middle class	Urban	T1 = 1-year, T2 = 2-years-old

Berry et al.	2012	United States	African-American and White	1292 Children	N/A	Lower income	Rural and semirural communities	Birth, 7-, 15-, 24-, 36-, and 60-months
Brand et al.,	2011	Switzerland	N/A	16 Infants	N/A	N/A	N/A	Newborns
Bright et al.	2014	United States	80% White	47 Toddlers	36% Girl	Middle income (Most parents with college degree)	N/A	12-, 18, 24-months
Burnham et al.,	2002	United States	76% White	80 Infants	47.5% Girl	Moderate to low SES	N/A	1-, 3-, 6-, 9-, 12-months
de Weerth et al.	2003	Netherlands	N/A	14 Infants	6 Girl, 8 Male	N/A	N/A	2- to 5-months-old
El-Sheikh et al.	2007	United States	69% White, 31% African American	166 Children	55% Girls	Diverse, mostly middle class	Rural and semirural communities	8- to 9-years-old
El-Sheikh et al.	2008	United States	66% White, 34% African American	64 Children	36 Girls and 28 Boys	Diverse, mostly middle class	Rural and semirural communities	8- to 9-years-old
Flom et al.	2007	United States	73% White, 11% Asian., 6.4 % Black, 3.7% Hsiapnic	90 mother-infant dyads	Not reported	High income	Urban	T1= 6-months-old; T2= 12-months-old

Galland et al.	2012	United States, Australia, Italy, Switzerland, China, UK, Hong Kong, Saudia Arabia, Japan, Canada, Taiwan, Netherlands, Russia, Israel, Spain, Korea, New Zealand	NA	34 papers with usable data (22 infant sleep, 7 toddler/preschool sleep, 13 child sleep)	NA	NA	NA	0-12 years
Henderson et al.	2011	NA	NA	26 Studies	NA	NA	NA	0-12 months
Hoyniak et al.	2019	United States	89% White	546 Toddlers	49% Girl	Middle Class	Urban	30-, 36-, & 42-months
Iglowstein et al.	2003	Switzerland	Swiss	493 Infants	261 boys and 232 girls	N/A	N/A	1, 3, 6, 9, 12, 18, and 24 months after birth and then at annual intervals

								until 16 years of age
Jian & Teti	2016	United States	87% White	139 families	51% Girl	Middle class (most college educated)	Urban	Infants 1, 3, and 6 months
Kerzevee et al.	2024	North America and Europe (15 cohorts, 16 studies)	Not consistently reported	1,904 infants; 17,079 salivary cortisol samples	Not specified	Not consistently reported	N/A	0–13 months
Kim & Teti	2014		84% White	106 mother–infant dyads	47% Girl	Majority of mothers were college-educated	N/A	1-, 3-, 6-, 9-months
Laurent et al.,	2016	UK	White British	100 mother-infat dyads	51% Girl	78% moms college educated; high SES	N/A	1-, 2-, and 3-years
LeBourgeois et al.	2013	United States	N/A	45 toddlers	44% Girl	Middle to upper-middle class	Urban	30–36 months
Lucas-Thompson et al.	2009	United States	52% non-Hispanic European American, 22% Hispanic American, 10% Asian/Asian American,	92 mother-infant dyads	49 boys (53%), 43 girls (47%)	Median family income $70,000-$80,000; 63% had less than 4-year college degree	Urban	6 and 12 months

			10% other ethnic groups					
McLaughlin et al.	2022	United States	100% African American	89 mother-infant dyads	47.19% Girls	68.5% mothers employed; 38.9% had bachelor's degree or higher	N/A	3 and 6 months
Mindell et al.	2016	United States	N/A	841	393 girls (46.7%) and 448 boys (53.3%)	Representative	N/A	Birth to 35 months
Mindell et al.	2015	Australia-New Zealand, Canada, China, Hong Kong, India, Japan, Korea, Malaysia, Philippines, Singapore, Thailand, United Kingdom, United States	N/A	10,085 Mother-child dyads	50.4% Girls	N/A	N/A	Birth through 5 years
Mindell et al.	2010	Multi-national (17 countries/regions)	N/A	29,287	51.9% boys, 48.1% girls	Higher education bias (83.7% had some	Urban	Birth to 36 months (grouped into 7 age

						college education);		categories: 0-2, 3-5, 6-8, 9-11, 12-17, 18-23, 24-36 months)
Mindell et al.	2013	Predominantly Asian countries/regions: China, Hong Kong, India, Korea, Japan, Malaysia, Philippines, Singapore, Thailand; Predominantly Caucasian countries: Australia, Canada, New Zealand, United Kingdom, United States	N/A	10,085 Mothers	49.6% Girls (children)	N/A	N/A	25 - 35 years old
Pecora et al.	2022	Italy	N/A	156	81 boys (51.9%), 75 girls (48.1%)	Middle SES	N/A	4 months and 8 months

Philbrook	2022	United States	80% White, 18% biracial, 2% Black	51	53% boys, 47% girls	Higher SES (69% bachelor's degree or higher)	Rural and semirural communities	3-6 years old (M=4.47 years, SD=0.89)
Philbrook et al.	2014	United States	84% White	167 Infants	53% girls	Higher SES (87% bachelor's degree or higher)	Rural and semirural communities	1- and 3-months
Philbrook & Teti	2016	United States	84% White	156 Infants	53% girls	Higher SES (87% bachelor's degree or higher)	Rural and semirural communities	3-, 6-, and 9-months
Räikkönen et al.	2010	Finland	Finnish	297 Children	53% girls	N/A	Urban	8-years-old
Sadeh et al.,	2009	United States and Canada	80% White	5006 infants and toddlers	48.12% Girls	Middle to Higher SES (87% with college degree or higher)	N/A	Birth to 36 -months
Saridjan et al.	2017	Netherlands	Dutch	322 Infants	42.5% Girls	Middle to Higher SES	N/A	12-20 months, 18-months, and 36-months
Scher et al	2010	Canada	70% White	27 toddlers	12 boys, 15 girls	Mixed (mother's education from some college to postgraduate degrees)	Urban	12-36 months (M=24.5 months)

Spruyt et al.	2008	Australia	N/A	20 infants	13 male, 7 Girl	Middle to Higher SES	Urban	Longitudinal study from birth to 12 months (assessments at 3, 6, 11, and 12 months)
Teti et al	2010	United States	91% White, 7% Asian American, 2% African American	45 families with infants (39 families for bedtime practices coding, 35 families for emotional availability coding)	23 girls, 22 boys	High (73% completed postsecondary education, 93% married, mean family income $61,323)	Rural and semirural communities	1-month, 3-month, 6-month, 12-month, and 24-month-olds
Tuladhar et al	2021	United States	62.2% White 13.3% Black or African American 7.8% Asian 4.4% Native American 12.2% Multiracial	86 healthy, singleton, 12-month-old infants and their parents	43 Girl, 43 male infants	Mixed socioeconomic status 42.4% economically strained (income-to-needs ratio <3) 79.9% mothers with 4-year college degrees 67.4% fathers with 4-year	Urban	Cross-sectional study with 12-month-old infants (M = 12.24 months, SD = 0.82)

						college degrees		
Voltaire & Teti	2018	United States	86.1% White	107 Families	N/A	Middle to Higher SES	Rural and semirural communities	1-, 3-, 6-, 9- months
Williamson et al	2021	United States	N/A	14,980 Mothers of young children	47.4% Girls	Lower SES	N/A	6-35.9 months
